# Magnetic microsphere-based portable solid phase extraction device for on-site pre-concentration of organics from large-volume water samples

**DOI:** 10.1038/s41598-017-08778-1

**Published:** 2017-08-14

**Authors:** Zhijian Yao, Qingqing Zhao, Yan Ma, Wei Wang, Qing Zhou, Aimin Li

**Affiliations:** State Key Laboratory of Pollution Control and Resource Reuse, School of the Environment, Nanjing University, Nanjing, 210023 P.R. China

## Abstract

In this research a new magnetic material called M88 was fully synthetized and characterized for the extraction of pharmaceutical and personal care products in water samples. In addition, a portable prototype of magnetic solidphase extraction (MSPE) device was developed for the onsite preconcentration. The MSPE coupling with high performance liquid chromatography-Diode array detector (HPLC-DAD) method was developed and validated for simultaneous analysis of 11 PPCPs (mefenamic acid, chloroamphenicol, ketoprofen, clofibric acid, indometacin, acetylsalicylic acid, bisphenol A, phenylphenol, gemfibrozil, triclosan, and ibuprofen) in environmental water samples. Experimental parameters affecting the extraction efficiencies, such as the amount of M88, desorption solvent, extraction time, and solution pH and sample volume were investigated. Under the optimal conditions, the limits of detection (LODs, S/N = 3) for the selected PPCPs were found to be in the range of 0.7–9.4 ng/L, with good linear correlation coefficients. It is also shown that the extraction efficiency of M88 was comparable to that of the commercial Oasis HLB and was evidently higher than that of the C18 cartridge. The optimised method was further verified by performing spiking experiments in water samples from Taihu Lake, with good recovery and reproducibility for all the compounds.

## Introduction

Over the years, various organic contaminants, such as pharmaceuticals and personal care products (PPCPs), have been frequently detected in water systems throughout the world^1–3^. PPCPs are recognized as a potential risk to aquatic ecosystems and human health in spite of their low environmental concentrations, which range from pg/L to ng/L^4^. Strict and accurate procedures must be conducted to monitor PPCPs to ensure that their concentration is below the safety level. However, quantifying PPCPs remains a significant challenge because of their low concentration, high polarity, and hydrophilic structures^5–7^. To address this issue, a sensitive and reliable pretreatment method is often required to enrich PPCPs in water.

Solid-phase extraction (SPE) is a widely-used sample pretreatment method^8^. But the high backpressure makes it time-consuming when handling water samples of large volume. Besides, the high cost and complex operation renders its applications in developing countries. In contrast, magnetic SPE (MSPE) has been accepted as a fast and simple approach which is able to preconcentrate trace organic contaminants in water samples^9–11^. Owing to the convenient sample preparation and the fast processing of large-volume samples^12^, MSPE can overcome the limitation of conventional SPE method.

As far as we know, the key factor that affects the performance of MSPE is the magnetic sorbent used. In the past few years, various magnetic sorbents have been synthetized for the concentration of PPCPs. For example, Magnetic hypercrosslinked polystyrene (HCP/Fe_3_O_4_), was prepared and used for preconcentration of four sulfonamides from natural water and milk samples^13^. Porphyrin-functionalized Fe_3_O_4_-graphene oxide (TCPP/Fe_3_O_4_-GO) nanocomposite was synthesized, used as a magnetic solid-phase extraction adsorbent for the preconcentration of seven sulfonamides (SAs) from environmental water samples^14^. Recently, our laboratory has also developed numerous of magnetic sorbents, which were capable of sorption of PPCPs such as tetracycline^15–17^, chlorotetracycline^18^, oxytetracycline^19^ and carbamazepine^12^.

It can be seen that the vast majority of such publications are focused on a single class of compounds. However, in the real environment, different classes of PPCPs are present in complex mixtures. As a result, magnetic materials that can achieve high extraction efficiency for a wide range of PPCPs must be developed, which are highly important to provide reliable knowledge about their occurrence, behaviour and ultimate fate in the environment^4, 20^. Simultaneous analysis of several groups of compounds with different physicochemical characteristics generally requires a compromise in the selection of experimental conditions, which in some cases means not obtaining the best performance for all compounds.

In addition, the transportation and storage of large-volume water samples are expensive and occasionally inconvenient, particularly in remote places. On site sample preparation process are always welcome in analytical chemistry field. However, the absence of available magnetic SPE material could clearly affect to the results reproducibility. Therefore, the development of a portable MSPE device and a usenable maganetic materal is urgent.

In this work, we aimed to develop not only a novel magnetic microsphere M88 with amino groups but also a portable MSPE device to realize the on-site pre-concentration of PPCPs with different polarity. To evaluate the performance and potential application of M88-based MSPE device, we conducted the pre-concentration of multiple PPCPs from large-volume aquatic environmental samples. The extraction conditions, including the eluting solution, amount of magnetic materials, extraction time, solution pH, and sample volume were systematically optimized. We also investigated the effect of humic acid on the extraction process, and analyzed several PPCPs in the real environmental water samples using our optimized methodology.

## Materials and Methods

### Materials

Standard Formic acid (FA), mefenamic acid (MA), chloroamphenicol (CP), ketoprofen (KEP), clofibric acid (CA), indometacin (IDM), acetylsalicylic acid (ASP), bisphenol A (BPA), 2-phenylphenol, gemfibrozil (GEM), triclosan (TCS), and ibuprofen (IBU) (>98% of purity for all the chemicals) were purchased from J&K Scientific (Shanghai, China). Methanol (Tedia, USA) and acetonitrile (ACN) (Tedia, USA) were HPLC-grade. Divinylbenzene (DVB, 80%), benzoyl peroxide (BPO, 98%), toluene (TOL, 99.5%), oleic acid (OA), and 1,2-dichloroethane (DCE, 99%) were provided by Sinopharm Chemical Reagent Co., Ltd. (China). Ferric chloride hexahydrate (FeCl_3_ • 6H_2_O), ferrous chloride tetrahydrate (FeCl_2_ • 4H_2_O), anhydrous ferric chloride (FeCl_3_), N,N-dimethyl-1,3-propanediamine, ammonium hydroxide, and other inorganic salts (analytical grade) were purchased from Nanjing Chemical Reagent Co., Ltd. (Nanjing, China). Ultrapure water prepared by the Milli-Q water purification system (Billerica, MA, USA) was used for all experiments.

### Preparation and characterization of magnetic hyper-crosslinked resin

OA-coated Fe_3_O_4_ nanoparticles were prepared by coprecipitating FeCl_3_ • 6H_2_O and FeCl_2_ • 4H_2_O with a molar ratio of two^12, 21^. At room temperature, FeCl_3_ • 6H_2_O and FeCl_2_ • 4H_2_O were dissolved in 500 mL of deionized water in a 1-L three-necked round-bottom flask and deoxygenated with a stirring speed of 500 rpm at N_2_ for 30 min. Subsequently, the mixture was heated to 353 K and continuously stirred in nitrogen atmosphere for another 30 min. Then, 50 mL of ammonia solution (25 wt–28 wt%) and 5 g of OA dissolved with 25 ml of acetone (AT) were added to the flask. After 10 min, another 5 g of OA was added dropwise to the reaction system. After 30 min, the OA-coated Fe_3_O_4_ nanoparticles were isolated using a strong magnet and washed five times with AT. Then, the magnetic nanoparticles were stored in nitrogen atmosphere.

Magnetic microspheres (M88) were synthesized by a three-step method, namely, suspension polymerization, amination, and sequential post-crosslinking (Figure S1). Initially, the oil phase contained OA-coated Fe_3_O_4_ nanoparticles (25 g), methylacrylate (25 g), DVB (75 g), TOL (150 g), and BPO (2.0 g). The mixture was stirred at 200 rpm and heated under a stream of nitrogen at 368 K for 12 h to acquire the magnetic polydivinylbenzene microspheres. The obtained microspheres were aminolyzed with N,N-dimethyl-1,3-propanediamine in a sealed pressure vessel. After rinsing and drying, the microspheres were fully swelled by DCE. Then, the catalyst FeCl_3_ (5 g) was introduced into the system, which was heated to 353 K for 12 h. The resultant magnetic microspheres (M88) were thoroughly washed five times with water and ethanol and dried at 333 K for 12 h under vacuum.

The morphology of M88 was examined by scanning electron microscopy (SEM, S-3400NII, Hitachi Japan). N_2_ adsorption/desorption experiments were conducted at 77 K to characterize the surface area and pore structure, which were calculated using the standard Brunauer–Emmett–Teller equation and Barrett, Joyner, and Halenda method, respectively. An accelerated surface and porosimeter system (Autosorb-iQ2, Quantachrome, USA) was employed for automatic calculation. The sample magnetization was characterized using a vibrating sample magnetometer (VSM, Quantum Design MPMS-5S).

### Portable magnetic solid phase extraction device

**Figure 1:**
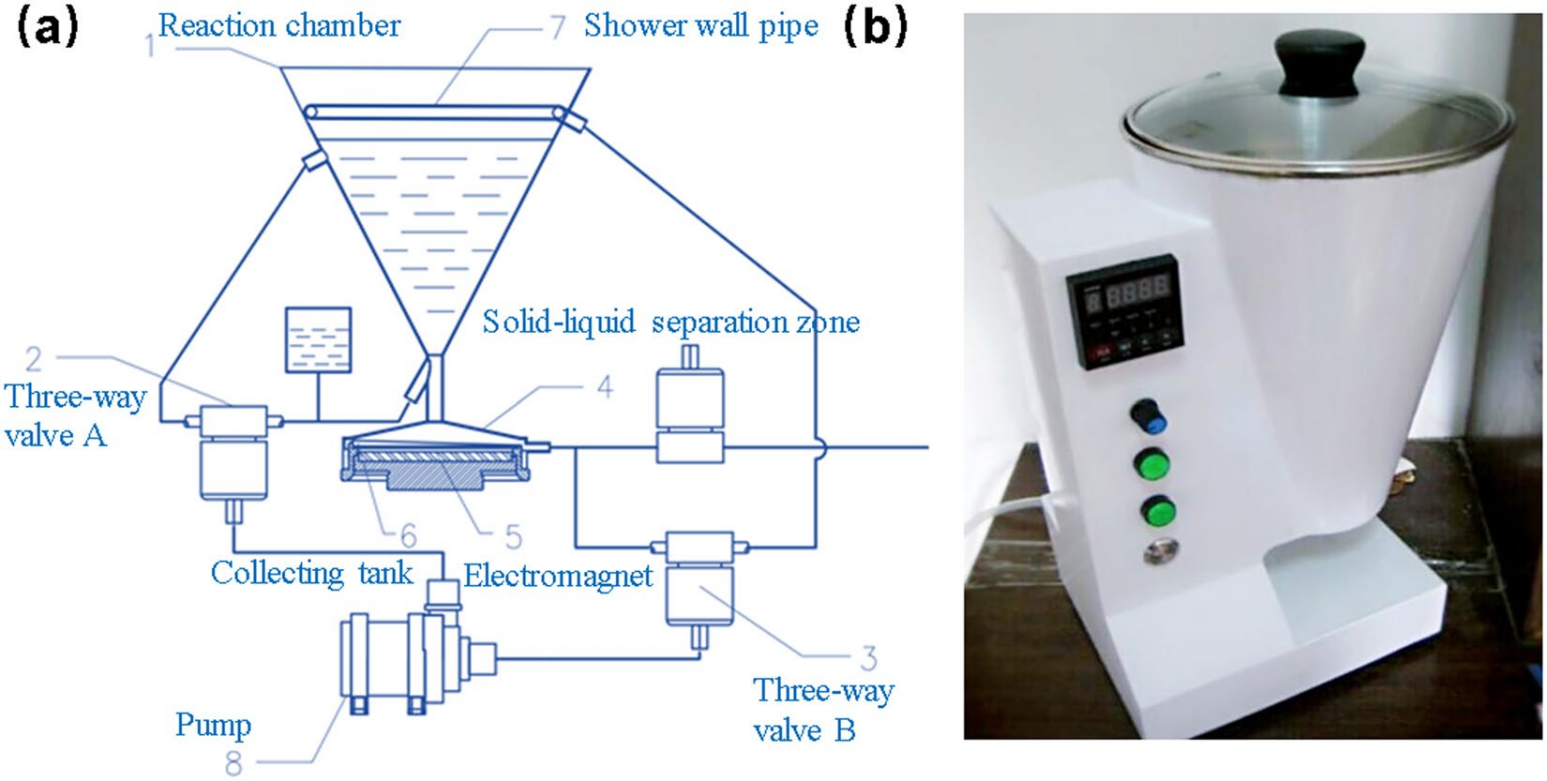
(**a**) Scheme and (**b**) Digital photo of the portable magnetic separator.

A portable reactor was designed to conduct on-site MSPE. Figure 1a shows the scheme of the prototype device. The structure contains five units: reaction unit, powder collection area, pump, shower wall pipe, and water tank. The reaction unit that has an inverted cone design was made of stainless steel, which facilitates the full mixing of water samples and magnetic agents. A round electromagnet was set in the powder collection area. When water passes through the collection area, the magnetic solid phase extraction agent is separated from the water body by the magnetic force and collected in a collecting groove, which is not magnetic. The MSPE agent on the groove is easily removed without magnetic force. The circulating pump is used for importing, exporting, and circulating water. The last important unit is the shower wall pipe and water tank, which were designed to collect the residual magnetic solid phase extraction agent in the pipe and ensure a high collection efficiency. Considering the demand of actual analysis and portability, the dimension was set to 190 cm (length) × 256 cm (width) × 404 cm (height), as shown in Fig. 1b. Furthermore, its weight was only about 5 kg, so it was suitable for on-site preconcentration application.

### MSPE operation procedure

**Figure 2:**
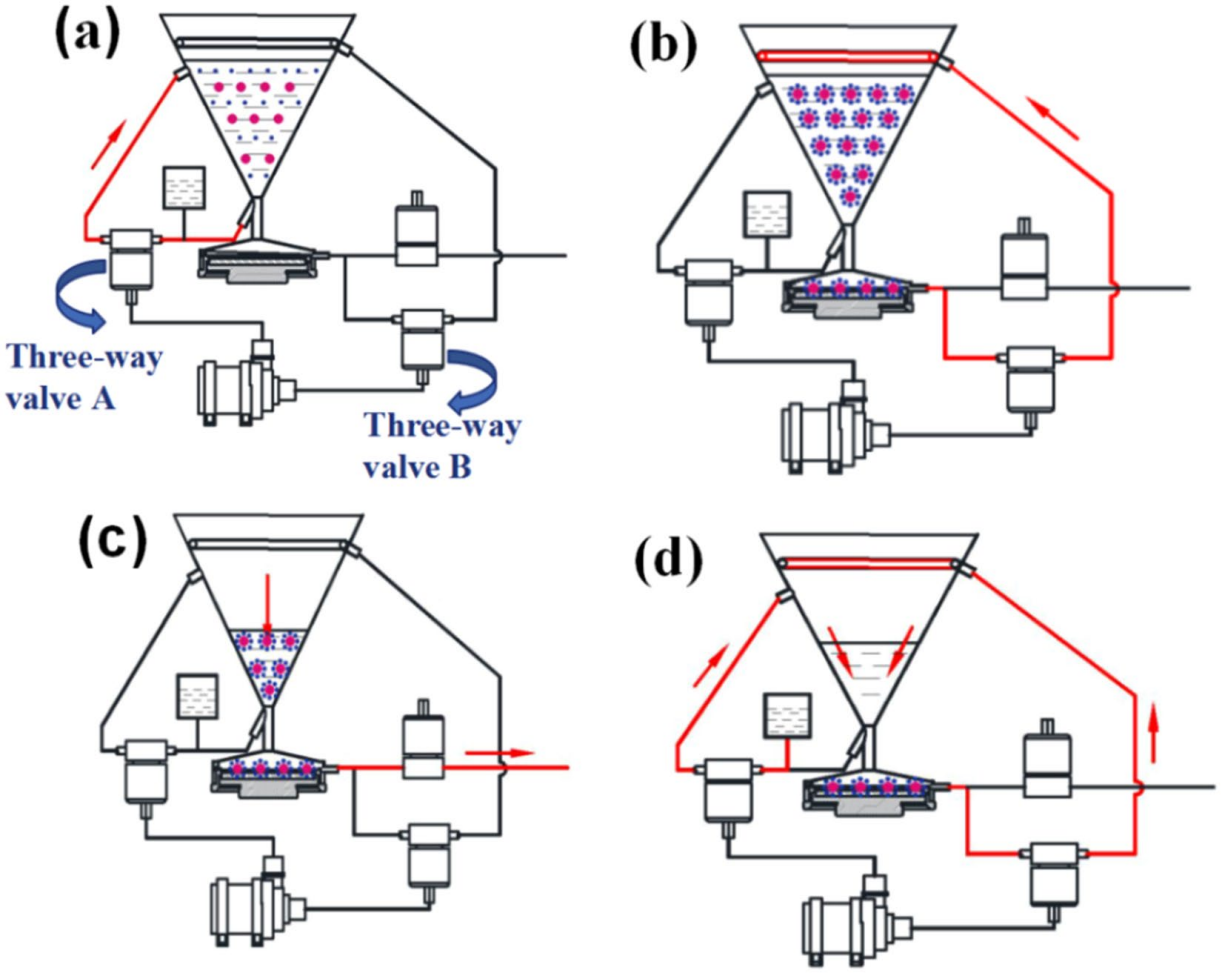
Procedure for magnetic solid phase extraction (MSPE). (**a**) circulated contact of M88 and PPCPs solution; (**b**) transfer of mixed solution of M88 and PPCPs into solid–liquid separation zone; (**c**) electromagnetic separation of M88 microsphere from mixed solution; (**d**) the transfer and collection of residual M88 by circulation washing.

The PPCPs were extracted using the designed portable device according to the following procedures, as shown in Fig. 2. First, 2.5 L of water samples spiked with certain amount of the selected PPCPs and 0.20 g of extraction agent M88 microspheres (activated) were added to the reaction chamber. The flow direction was controlled by three-way valve system. To ensure sufficient contact, the solution and M88 were circulated by a pump at a flow rate of 16 L/min for 30 min with three-way valve A open (Fig. 2a). After extraction, three-way valve A was closed, and valve B and the outlet were opened. The mixed solution of M88 and PPCPs was then transferred into the solid–liquid separation zone (Fig. 2b). M88 microspheres were collected in the collecting tank using an electromagnet. The solution was separated automatically and transferred to a shower wall pipe via the three-way valve B (Fig. 2c). Through the circulation washing of the shower wall pipe, the residual M88 in the reaction chamber was also transferred into the solid–liquid separation zone and collected (Fig. 2d). Finally, the electromagnet was switched off, the collecting tank was taken out, and M88 was transferred to the elution device. The extraction process was completed automatically on site. Subsequently, M88 with adsorbed PPCPs was washed with methanol (5%) to release the nonspecific adsorption and dried under a nitrogen stream. The compounds adsorbed on the microspheres were eluted with 10 mL of 0.75% FA-ACN solution. The eluate was dried with nitrogen, and the residue was reconstituted with 0.5 mL of ACN. Finally, the solution was filtrated and analyzed by HPLC. The recoveries were calculated by the ratio of the concentration added and the concentration detected after the extraction.

### HPLC analysis

The adsorbed target compounds were analyzed by high-performance liquid chromatography (HPLC). Chromatographic measurement was performed using an Agilent 1200 system with a Diode array detector (DAD). The DAD detection wavelength were 205 nm for CA, IB, GEM, OPP, ASP, 260 nm for KF, IDM and 280 nm for CP, MA, TCS, BPA respectively. Separation was conducted on an Agilent Eclipse XDB-C18 column (4.6 × 250 mm, 5 μm). Gradient separation was performed using a 0.1% H_3_PO_4_ aqueous solution and ACN as the A and B solvents see (Table S1). Concentration and velocity gradients were taken to separate the 11 target compounds. The linear gradient profile was as follows: the concentration of B was initially maintained at 35%, the concentration of B was linearly increased to 55% over a period of 5.5 min, and the concentration of B was maintained over a period of 33 min. The flow rate was initially maintained at 1 mL/min, the flow rate was linearly decreased to 0.5 mL/min at 6 min and maintained over a period of 22.5 min, the increased to 1 mL/min again at 23.5 min. The column temperature is set as 30 °C.

## Results and Discussion

### Characterization of the amino-modified magnetic hyper-crosslinked resin

**Figure 3:**
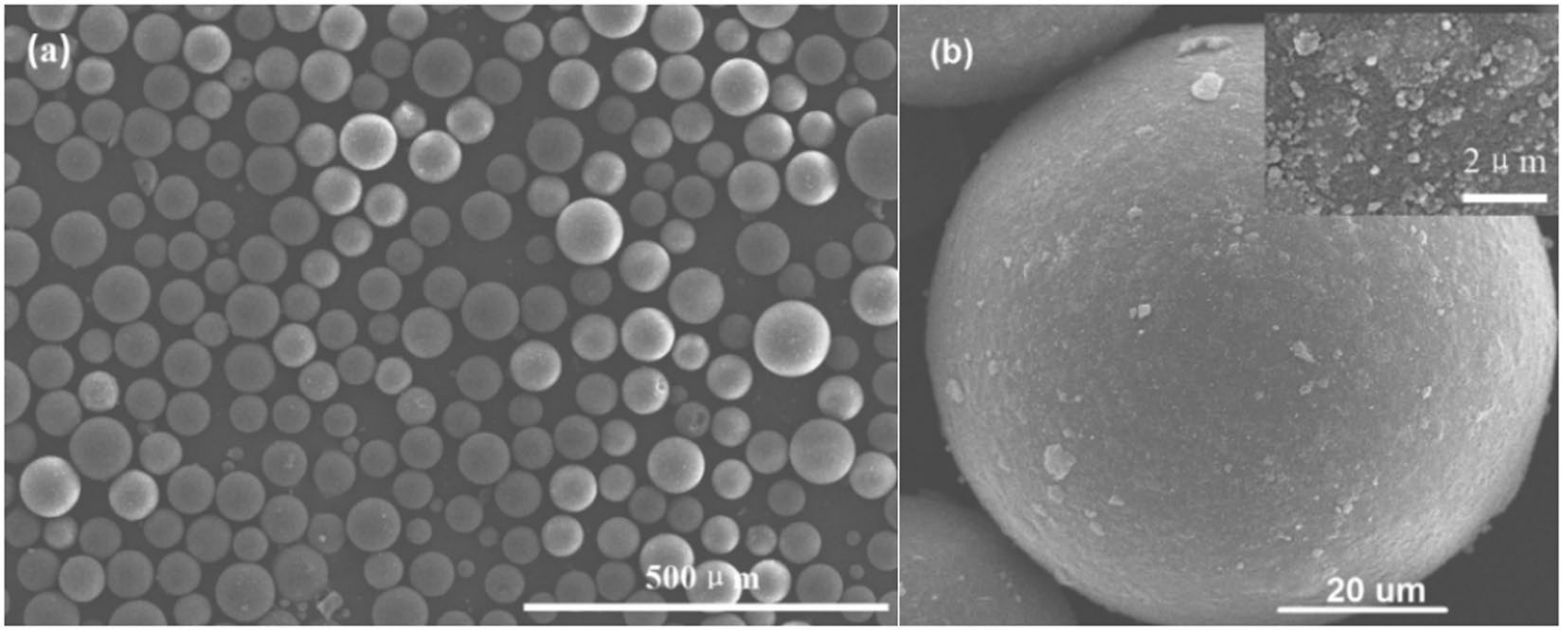
(**a**) Low magnification and (**b**) high magnification SEM images for M88.

**Figure 4:**
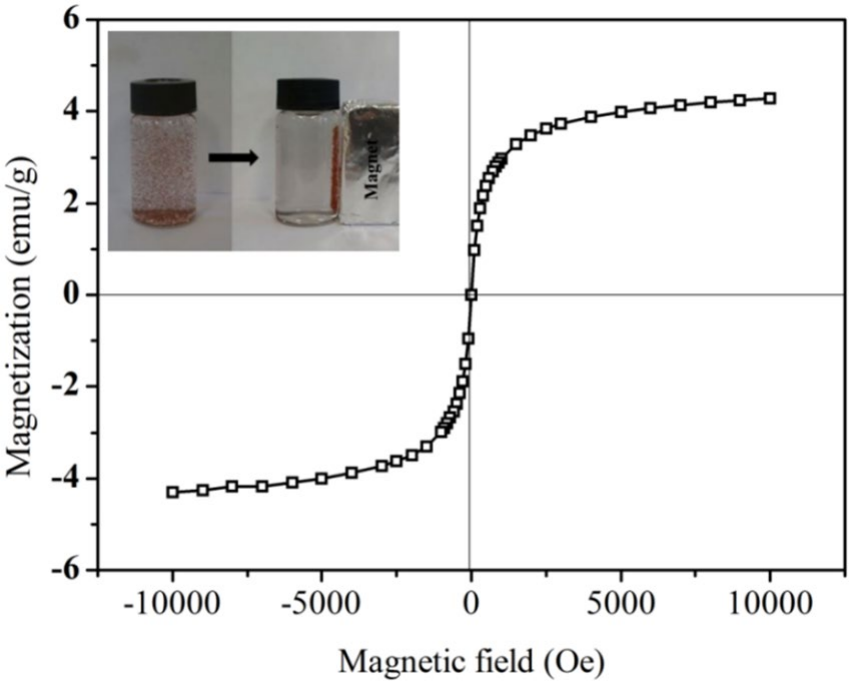
Magnetization curve of M88 and the separation of M88 from solution by a magnet.

SEM was conducted to analyze the surface and morphology of amino-modified magnetic hyper-crosslinked resin (M88). Figure 3a shows that M88 has a regular spherical shape with a smooth surface. The diameter of M88 microspheres is 55–85 µm. The rough surface of M88 (Fig. 3b) can be attributed to the successful addition of magnetic particles. Figure 4 shows that the saturation magnetization of M88 reached as high as 4.2 emu/g. M88 could be evenly dispersed in a solution for adsorption and easily separated from a solution using a magnet. These are advantages that ensure the potentials of M88 in the adsorption, separation, and regeneration of PPCPs^22^.

The textural and physicochemical properties of M88 are listed in Table 1. The surface area and total pore volume of M88 reach as high as 796 m^2^/g and 0.80 cm^3^/g, respectively. The pore structure of M88 is dominated by mesopores with an average pore size of 4.00 nm. More importantly, M88 has a high weak base anion exchange capacity (1.32 mmol/g), which could provide sufficient adsorption sites to obtain PPCPs^22, 23^.

**Table 1:** Textural and physicochemical properties of M88.

BET specific surface area (m^2^/g)	weak base exchange capacity (mmol/g)	high base exchange capacity (mmol/g)	Average pore diameter (nm)	Micropore volume (cm^2^/g)	Mesopore volume (cm^2^/g)	Pore volume (cm^2^/g)
796	1.32	0.84	4.00	0.06	0.61	0.80

### Optimization of experimental conditions

To achieve the optimal recovery for extraction of 11 target PPCPs in water, the MSPE conditions, including elution solvent, solution pH, amount of the sorbent, extraction time and volume of the sample solution, were thoroughly optimized following the MSPE procedure described above. All experiments were performed in triplicate.

### Effect of desorption solvent

**Figure 5:**
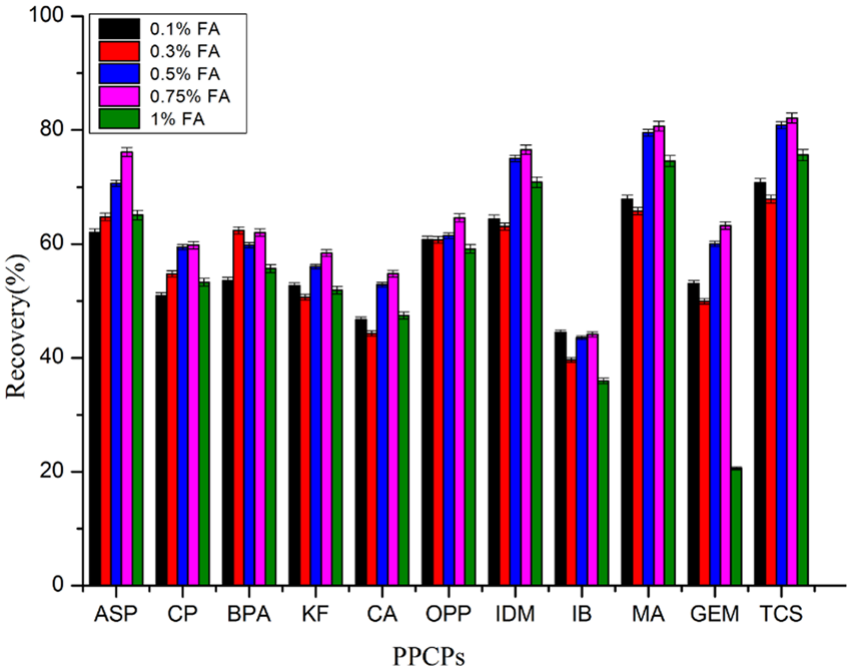
Recovery for the extraction of 11 target compounds (CP, ASP, CA, BPA, KF, OPP, IDM, IB, MA, and TCS) using a mixture of FA and ACN at different ratios.

MeOH, ACN, ethyl acetate, and acetone were employed as eluents to test for the elution recovery of PPCPs from M88, which were spiked at 100 ng/L for each compound. As shown in Figure S2, ethyl acetate exhibited the lowest recovery for each compound, which may be attributed to the relatively polar nature of these PPCPs. Better recoveries were obtained with MeOH, ACN and acetone as the elution solvents. The best recoveries were achieved with elution by ACN. Thus, ACN was chosen as the elution for the simultaneous extraction of all the selected PPCPs. This is probably because of its high dielectric constant of 38.8 and dipole moment of 3.92 D. To achieve a higher recovery, the effect of mixture of FA and ACN at different volume ratios (0.1–1%) on the desorption efficiency of the target compounds was further investigated. Figure 5 shows that the addition of FA into the desorption solvent could increase the extraction efficiency of the target compounds. For example, the recovery of MA increased from 50% to 80% when 0.75% FA was added. Increasing the amount of FA further to 1% decreased recovery. This phenomenon maybe attributed to that the addition of excessive amount of FA probably induces the transformation of certain PPCPs from the ionic state to the neutral state, weakening the ion exchange interaction. Accordingly, the optimum ratio of FA to ACN was 0.75%, which was used in the following experiments.

### Effect of M88 amount

**Figure 6:**
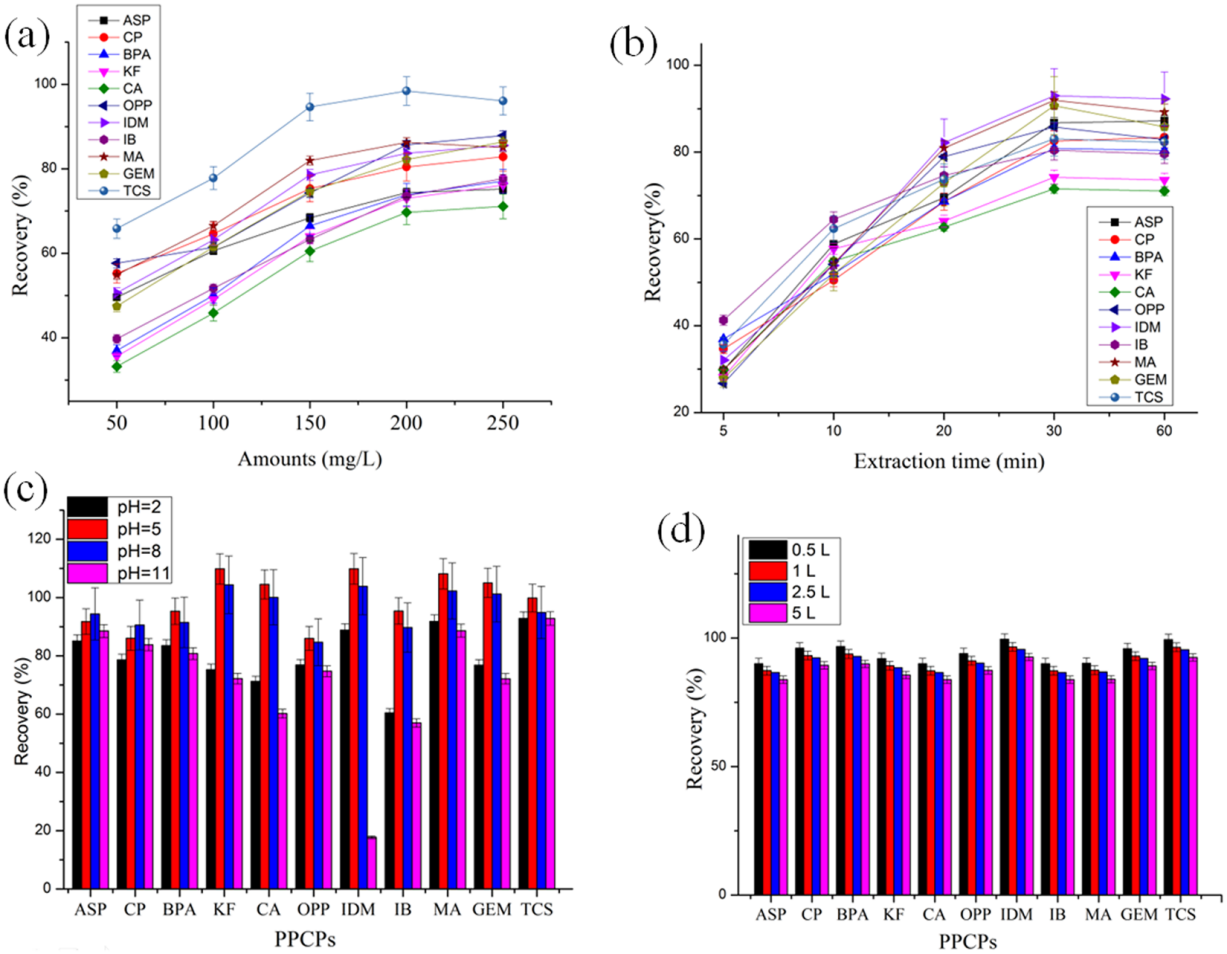
Recovery for the extraction of the 11 target compounds: (**a**) effect of extraction agent amount, (**b**) effect of extraction time, (**c**) effect of solution pH, and (**d**) effect of sample volume.

The amount of M88 influences extraction performance significantly. Figure 6a shows that the recoveries of the eleven PPCPs were increased with increasing the amount of sorbent from 50 mg/L to 150 mg/L. A high dosage of M88 can provide additional adsorption sites for PPCPs. The recovery slightly increased or was comparable to that using 150 mg/L when the amount of M88 was increased to 200 mg/L. This result indicated that excessive M88 cannot promote recovery significantly. This is probably because the organic compounds already or nearly reached their saturated adsorption^24^. Therefore, 200 mg/L was adopted for further analysis.

### Effect of extraction time and sample pH

The effects of extraction time and solution pH were also investigated to determine the optimal extraction conditions. Figure 6b shows that the recovery only reached approximately 25–35% at an extraction time of 10 min. At a longer extraction time (30 min), recovery increased to 65–90%, which remained the same when the extraction time was extended to 60 min. This result indicated that the adsorption of PPCPs almost reached equilibrium within 30 min. The rapid extraction process was attributed to the high surface area, relatively uniform mesoporous structure, and the high weak base anion exchange capacity of M88.

The pH value of the solution also controls the recovery because the presenting status of the 11 PPCPs may vary from the neutral to the ionic form if the medium has various pH values, thus leading to different interactions between the M88 and the PPCPs. The effect of pH on the extraction efficiency was investigated by adjusting the pH value from 2 to 11. The results (Fig. 6c) show that the recovery of ASP, CP, BPA, OPP, MA, and TCS remained the same regardless of pH changes because of the neutral form of these pollutants throughout the studied pH range. By contrast, the recovery of KF, CA, IDM, IB, MA, and GEM improved from 60–70% to 85–100% when the pH was increased to 8. However, increasing the pH further to 11 decreased the recovery to 20–60%. The low recovery for all the PPCPs under strong acid and alkali conditions was probably because the rich H^+^ or OH^−^ ions can form an electrostatic layer between the surface of the extraction material and the target molecules. This barrier probably hindered the adsorption, diffusion, and contact of target PPCPs towards the extraction material^18, 21^. At pH 5, the recovery was the highest for the multiple organics. This is probably because of the combination of both the high specific surface area and the strong ion exchange capacity. At pH 5, the surface oxygen- and nitrogen-containing groups of M88 can be protonated to form hydroxonium and ammonium moieties, which can facilitate the interaction with adsorbates through hydrogen bonds^25, 26^.

### Effect of sample volume

With the aim of reducing LODs, the effect of sample volume on extraction was investigated and optimized. Ultrapure water samples ranging between 0.5 and 5 L were spiked with 100 ng/L of each target compound and were pre-concentrated following the MSPE procedure described above. It is clear from Fig. 6d that although the recoveries are reduced when the sample volume is increased from 0.5 L to 5 L, all the values still remain above 80%. As it can be seen, the recoveries are in the range of 90.0–99.4%, 87.3–96.6%, 85.5–94.6% and 82.8–91.6% for 0.5, 1, 2.5 and 5 L respectively. The results show that M88 has the potential to endure a large volume of solution. Therefore, a sample volume of 2.5 L was selected as a compromise between LODs and recoveries, in addition to portability.

Through all the analyses, the optimum extraction condition, solution pH, and extraction time were determined to be 200 mg/L M88, pH 5, and 30 min for a 2.5 L solution, respectively.

### Performance comparison between M88 and commercial sorbents

**Figure 7:**
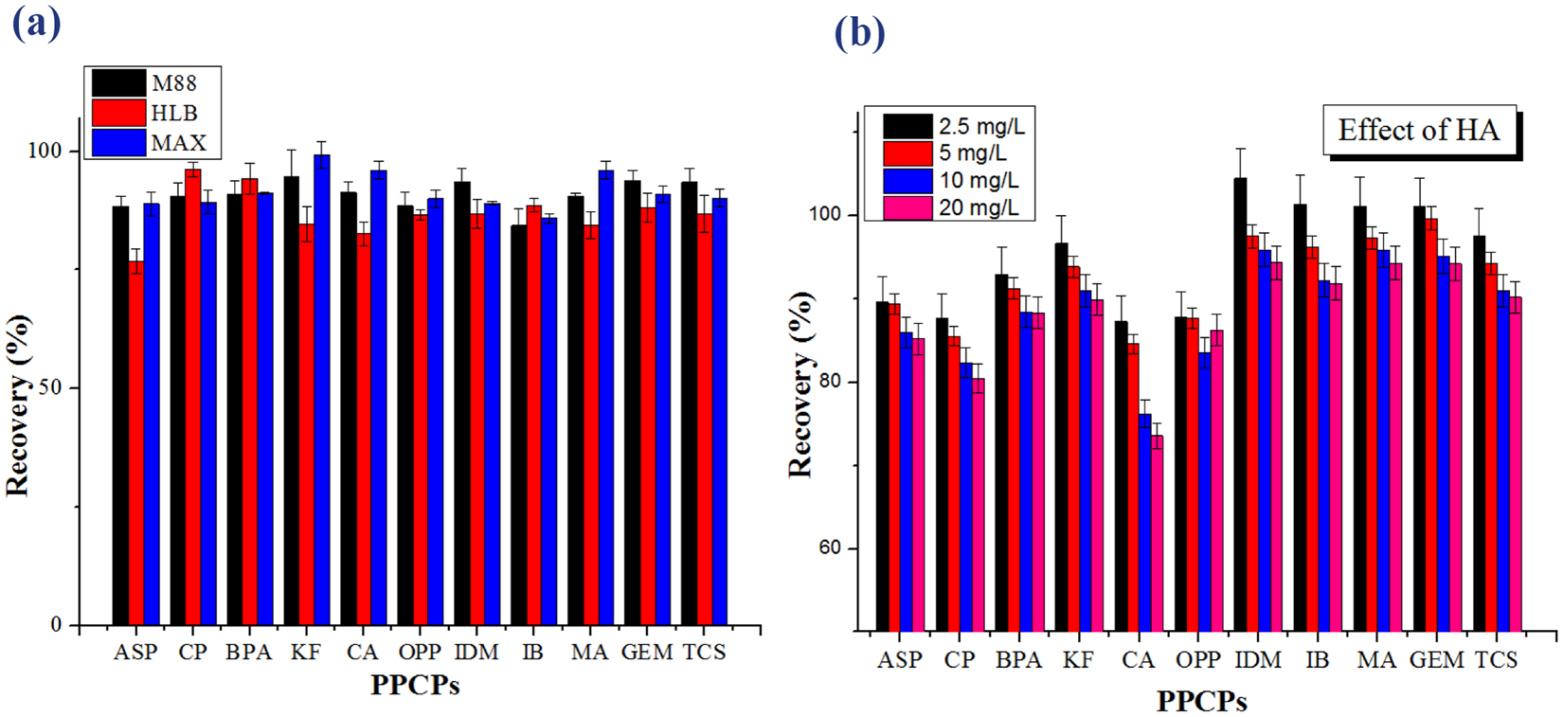
(**a**) Comparison of M88 performance with commercial extraction materials. (**b**) Effects of HA on the recovery of PPCPs by M88.

Oasis HLB and MAX are commercially available SPE sorbents, which are widely used in the preconcentration of PPCPs from domestic sewage, surface water, and groundwater^27–29^. Figure 7b shows the recoveries of PPCPs in real water samples by the three extraction materials. In general, M88 exhibits better extraction efficiency than HLB, and is comparable to MAX. The recoveries of CP, BPA as well as IB with HLB are slightly higher than that with M88 or MAX. On the contrary, M88 and MAX performed better extraction efficiency for other eight target compounds, which may result from the electrostatic interaction between the acidic functional groups on PPCPs and the amino group on M88 or MAX. As can be seen, five out of the eight target compounds are acidic, with pKa values between 3.5 and 4.9. Thus, M88 and MAX are more suitable than HLB for the extraction of acidic compounds. Extraction time must also be considered in addition to the extraction performance. The extraction time for the proposed method was around 30 min, which is approximately 5 times faster than the conventional tube-SPE procedure^30^. This result demonstrates that M88 is a promising extraction material which can be used for the extraction of PPCPs from environmental water.

### Effect of humic acid

An actual water system generally contains complex natural organic matters (NOMs). Studying the extraction performance in the presence of NOM is necessary to apply the novel magnetic M88 microspheres to pretreat actual water samples. The influence of humic acid (HA), a typical NOM, on the extraction behavior of M88 was investigated. Figure 7b shows the extraction efficiency of PPCPs in the presence of HA at different concentrations. Within a wide concentration range of 2.5–20 mg/L, the presence of HA did not exert significant influence on the extraction performance of the selected target compounds, except CA. The recovery of CA remained the same at low HA concentrations (2.5–5 mg/L), whereas the recovery decreased from roughly 85% to approximately 75% at higher HA concentrations (10–20 mg/L). With a pKa value of 2.9, CA tends to be ionized at a high degree. Therefore, the adsorption of CA on the surface of M88 was dominated by the ion-exchange interaction, which suffered from severe competition with the negatively-charged HA. The HA concentrations in most water samples range from 1 mg/L to 5 mg/L^12, 31, 32^. The aforementioned results demonstrated that HA at a low concentration exhibited a negligible effect on the recovery of PPCPs. Therefore, the M88 materials can be potentially used to extract PPCPs from most water samples.

### Analysis performance

The MSPE procedure based on M88 combined with HPLC was established to analyze the 11 target compounds. The calibration curves for the extraction of the 11 target compounds were obtained by plotting the HPLC signals (peak areas) against the initial concentrations in aqueous solution prior to MSPE. Table 2 shows the results obtained for the standards prepared in the MSPE extract of a river water sample. The LODs and limits of quantification (LOQs) were calculated as the concentrations corresponding to the signals of 3 and 10 times the standard deviation of the baseline noise, respectively. The preconcentration factor for the 11 target compounds was approximately 5000-fold. The LODs and LOQs were 3.8–20.8 ng/L and 12.7–69.2 ng/L, respectively. As observed, the proposed method, based on the employment of M88 as a magnetic sorbent, exhibited high efficiency for extraction of PPCPs from large-volume environmental water samples at trace levels. Instrumental precision was evaluated through repeatability injections (0.5, 2.0, and 5.0 µg/mL). Thus, the relative standard deviations (RSDs) were lower that 10% and no carry-over was observed. These results suggested that this MSPE-HPLC detection method was feasible and reliable.

**Table 2:** Analysis performance for the determination of eleven target compounds in environmental water using MSPE-HPLC.

PPCPs	Linearity range (µg/mL)	Calibration equations	R^2^	River water MSPE extract	
				LOD^a^ (ng/L)	LOQ^b^ (ng/L)
CP	0.05–5.0	y = 22.888x − 1.769	0.9984	18.2	60.8
ASP	0.05–5.0	y = 43.398x − 7.332	0.9978	15.4	51.2
BPA	0.05–5.0	y = 11.568x − 12.798	0.9990	4.3	14.4
KF	0.05–5.0	y = 38.135x + 0.508	0.9986	5.8	19.4
CA	0.05–5.0	y = 24.081x − 3.215	0.9995	8.9	29.6
OPP	0.05–5.0	y = 25.889x − 7.979	0.9989	3.8	12.7
IDM	0.05–5.0	y = 27.319x + 5.215	0.9953	8.4	28.0
IB	0.05–5.0	y = 25.923x + 8.338	0.9960	20.8	69.2
MA	0.05–5.0	y = 16.038x + 7.231	0.9961	8.1	27.0
GEM	0.05–5.0	y = 36.139x − 9.783	0.9990	8.8	29.2
TCS	0.05–5.0	y = 45.633x − 6.069	0.9989	6.7	22.4

^a^Detection limits are calculated using S/N = 3. ^b^Quantification limits are calculated using S/N = 10.

### Application to environmental water samples

**Figure 8:**
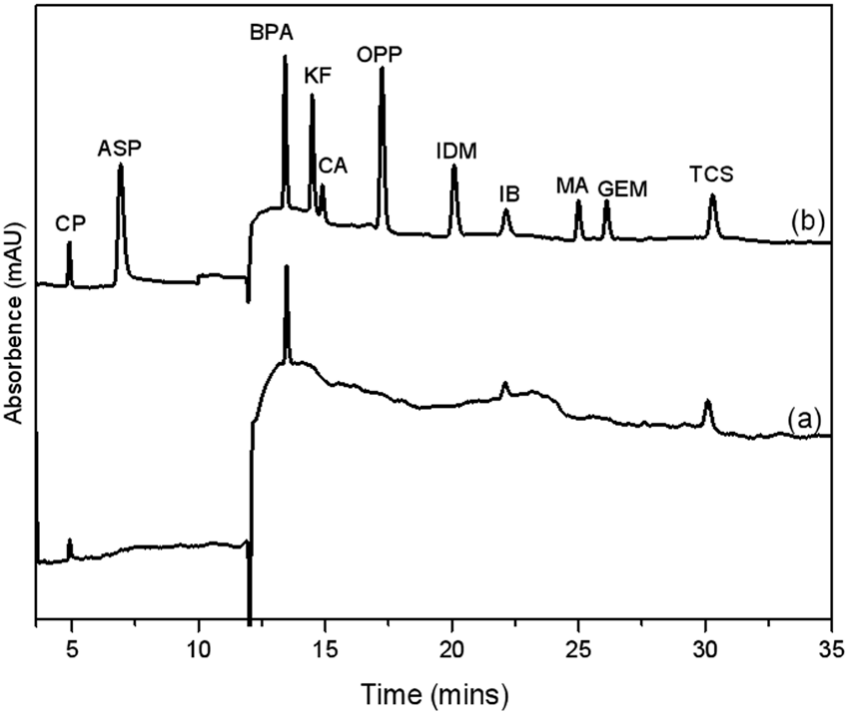
Typical chromatograms of the blank river water sample (**a**), the blank river water sample spiked with 0.1 ng/mL of PPCPs (**b**).

To demonstrate the applicability of the proposed MSPE-HPLC detection method, we analyzed the selected PPCPs in actual environmental water samples, which were taken from Gong Bay, a vital surface water source in the Taihu region (Jiangsu Province, China). The MSPE processes were conducted on site. Figure 8 indicates that CP, BPA, IB, and TCS were detected in the lake water samples (Fig. 8a) with concentrations of 0.029, 0.033, 0.043, and 0.016 ng/mL, respectively (Table 3). The high detection level of IB can be attributed to its frequent use in China with an annual production of over 1000 tons^33^. The recoveries of the selected compounds were investigated by spiking the Taihu Lake samples with standard solutions with concentrations of 0.05 ng/mL and 0.1 ng/mL. The relative recoveries, which were expressed as the mean value (n = 3), ranged from 81.0% to 104.9% with a RSD between 3.5% and 9.7%, as shown in Table 3. The on-site preconcentration of the proposed method, which was more convenient and economical than conventional tube-SPE, took approximately 30 min. The MSPE method we developed possessed the advantages of low limits of detection, high extraction efficiency, as well as fast and easy operation. However, the limitation of the method lies in that the method is preferred for the preconcentration of acidic compounds.

**Table 3:** Determination of eleven PPCPs and their recoveries from river water samples (n = 3).

PPCPs	River water (2.5 L)			
	Spiked (ng/mL)	Detected (ng/mL)	Recovery (%)	RSD (%)
ASP	—	n.d.	—	—
	0.05	0.046	88.3	7.1
	0.10	0.090	90.4	5.3
CP	—	0.029	—	5.4
	0.05	0.074	89.2	9.1
	0.10	0.116	87.1	8.4
BPA	—	0.033	—	6.3
	0.05	0.079	91.3	7.2
	0.10	0.124	90.8	5.1
KF	—	n.d.	—	—
	0.05	0.051	101.4	6.4
	0.10	0.104	104.6	5.3
CA	—	n.d.	—	—
	0.05	0.047	95.1	6.3
	0.10	0.096	96.3	5.2
OPP	—	n.d.	—	—
	0.05	0.043	85.4	8.3
	0.10	0.082	82.0	5.3
IDM	—	0.040	—	4.5
	0.05	0.089	97.8	8.6
	0.10	0.145	104.6	5.2
IB	—	0.043	—	6.5
	0.05	0.095	105	5.3
	0.10	0.134	90.9	3.5
MA	—	n.d.	—	—
	0.05	0.046	92.5	7.6
	0.10	0.103	103.0	6.3
GEM	—	n.d.	—	—
	0.05	0.049	98.2	5.2
	0.10	0.100	100.1	4.3
TCS	—	0.016	—	9.7
	0.05	0.065	97.7	8.6
	0.10	0.111	95.1	7.4

## Conclusion

We synthesized amino-modified magnetic hyper-crosslinked microspheres M88. These microspheres have a large specific area of 796 m^2^/g, uniform mesopores, and a high weak base exchange capacity of 1.32 mmol/g. Coupling with a portable MSPE device, the magnetic microsphere exhibited excellent performance for extracting multiple PPCPs simultaneously on-site with a short extraction time (~30 min). Even with the presence of humic matter (2–20 mg/L), M88 still showed an outstanding performance in extracting the selected PPCPs (>80% recovery) with negligible interference. The proposed MSPE-HPLC based on M88 was proven effective for analyzing actual water samples. This newly designed integrated method will be easily applied in actual water systems because on-site sample pre-concentration is easily operated and economical, and detection is reliable and highly sensitive.

## Electronic supplementary material

Supplementary Information
