# Supplementary material for: Magnetic microsphere-based portable solid phase extraction device for on-site pre-concentration of organics from large-volume water samples
Source: Sci Rep. 2017 Aug 14;7:8069. doi: 10.1038/s41598-017-08778-1 (PMC5556110; doi:10.1038/s41598-017-08778-1)
Supplement: Supplementary file 1 — Supplementary Information [file 41598_2017_8778_MOESM1_ESM.doc]

**Supporting information**

**Magnetic microsphere-based portable solid phase extraction device for on-site pre-concentration of organics from large-volume water samples**

Zhijian Yao, Qingqing Zhao, Yan Ma, Wei Wang, Qing Zhou*, Aimin Li*

State Key Laboratory of Pollution Control and Resource Reuse, School of the Environment, Nanjing University, Nanjing 210023, P.R. China

**6 pages, 2 table, 2 figures**

Corresponding authors:

Qing Zhou, Ph.D., Associate Professor.

E-mail: [zhouqing@nju.edu.cn](mailto:zhouqing@nju.edu.cn)

Aimin Li, Ph.D., Professor.

E-mail: liaimin@nju.edu.cn

Tel.: +86-25-89680379. Fax: +86-25-89680377.

**Table S1**. HPLC mobile phase and flow velocity gradient

| Time（min） | Function | Parameter |
| --- | --- | --- |
| 0.00 | solvent component | A：65.0% B：35.0% |
| 5.10 | solvent component | A：65.0% B：35.0% |
| 5.50 | solvent component | A：45.0% B：55.0% |
| 5.50 | flow | 1ml/min |
| 6.00 | flow | 0.5ml/min |
| 22.50 | flow | 0.5ml/min |
| 23.50 | flow | 1ml/min |
| 33.00 | solvent component | A：45.0% B：55.0% |
| 34.00 | solvent component | A：65.0% B：35.0% |

**Table S2**. Physical and chemical properties of the target compounds.

| Compound | Formula weight | pKa | logK_ow_ | Molecular structure |
| --- | --- | --- | --- | --- |
| Chloramphenicol | 323.13 | 11.0 | 1.1 | 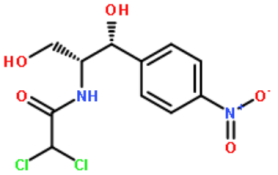 |
| Ketoprofen | 254.28 | 4.5 | 3.12 | 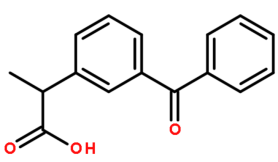 |
| Clofibric acid | 214.64 | 2.9 | 2.57 | 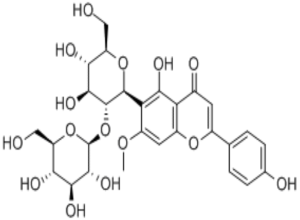 |
| Indomethacin | 357.79 | 4.5 | 4.27 | 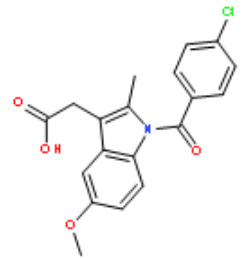 |
| Ibuprofen | 206.2 | 4.9 | 3.97 | 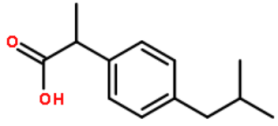 |
| Mefenamic acid | 241.29 | 4.2 | 5.12 | 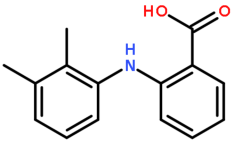 |
| Gemfibrozil | 250.33 | 4.7 | 4.77 | 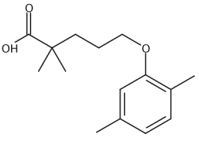 |
| 2-phenylphenol | 170.21 | 10.0 | 3.09 | 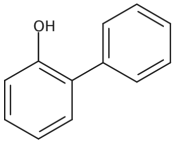 |
| aspirin | 180.16 | 3.5 | 1.19 | 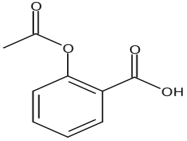 |
| triclosan | 289.54 | 7.8 | 4.76 | 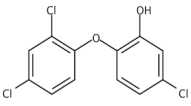 |
| Bisphenol A | 228.29 | 10.3 | 3.32 | 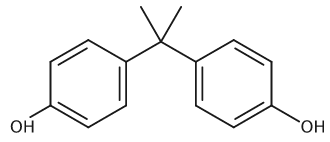 |

**Figure S1.** Schematic illustration of the synthesis of amino-modified magnetic hyper-crosslinked microspheres (M88).

**Figure S2.** Selecting of eluting solvent.
